# Factors Associated with the Acceptability of Mass Drug Administration for Filariasis: A Systematic Review

**DOI:** 10.3390/ijerph191912971

**Published:** 2022-10-10

**Authors:** Ahmad Farid Nazmi Abdul Halim, Dzulfitree Ahmad, Jane Ling Miaw Yn, Noor Azreen Masdor, Nurfatehar Ramly, Rahayu Othman, Thinakaran Kandayah, Mohd Rohaizat Hassan, Rahmat Dapari

**Affiliations:** 1 Department of Community Health, Faculty of Medicine, Universiti Kebangsaan Malaysia, Kuala Lumpur 56000, Malaysia; 2 Borneo Medical and Health Research Centre, Faculty of Medicine and Health Sciences, Universiti Malaysia Sabah, Kota Kinabalu 88400, Malaysia; 3 Department of Community Health, Faculty of Medicine and Health Sciences, Universiti Putra Malaysia, Serdang 43400, Malaysia

**Keywords:** acceptability, elephantiasis, lymphatic filariasis, mass drug administration

## Abstract

Mass drug administration (MDA) has been implemented as a tool to eliminate lymphatic filariasis. Acceptability among susceptible populations is crucial to achieving MDA effective coverage. This systematic review aims to present and systematically determine the factors associated with the acceptability of MDA. Articles related to factors associated with acceptability were collected electronically from three different databases (Scopus, Web of Science, and PubMed). Four pairs of independent reviewers screened the titles and abstracts of the collected data, stored in EndnoteX7, against the inclusion criteria. Afterwards, the included articles have been critically appraised to assess the quality of the studies using the Mixed Method Appraisal Tool (MMAT). Of the 68 articles identified, 11 were included in the final review. Knowledge, awareness, attitude and perceptions, communications, delivery and accessibility of MDA, gender, and age are the factors associated with MDA acceptability. Community acceptance remains a challenge in the implementation of MDA. To expand MDA coverage in all endemic countries, there is a strong need to address the factors influencing community acceptance of MDA.

## 1. Introduction

Lymphatic filariasis (LF), a vector-borne disease, is caused by three species of parasitic worms, namely, *Wuchereria brancofti*, *Brugia malayi*, and *Brugia timori*, which are transmitted by mosquitoes [1]. LF is one of the most disfiguring diseases in the world: it causes permanent disability leading to social stigma as well as economic loss. Even though LF is not a deadly disease, the affected person may have to live with the disability throughout their lives. Globally, over 120 million people were infected in 2000, with approximately 40 million disfigured and disabled by the disease. Almost 863 million people in 47 countries worldwide remain threatened by LF and require preventive chemotherapy to stop the spread [2]. In response, the World Health Organization (WHO) launched the Global Program to Eliminate Lymphatic Filariasis (GPELF) in 2000 with two main objectives, to interrupt the transmission of LF as well as to alleviate suffering and decrease the disability caused by LF [3].

Mass drug administration (MDA) was adopted as a tool for disrupting the transmission of LF. Antifilarial treatments were administered to all eligible community members residing in an endemic area regardless of their infection status. This preventive chemotherapy consisted of an annual single dose of a combination of two drugs (diethylcarbamazine and Albendazole (DA) in communities without onchocerciasis or ivermectin and Albendazole (IA) in communities where LF and onchocerciasis are co-endemic) administered for a minimum of five consecutive years to the entire eligible population living in endemic areas [4]. In 2017, a three-drug regimen comprising ivermectin, diethylcarbamazine and Albendazole (IDA) was introduced by the WHO as an alternative MDA regimen to accelerate the LF elimination program [5].

MDA helps in primary prevention by lowering and reducing transmission rates among at-risk populations. Furthermore, MDA can prevent the progression of subclinical to clinical disease and deteriorating morbidity [5], contributing to economic savings at the community level. The effectiveness of MDA in reducing the prevalence and density of microfilaria in the blood is directly related to the proportion of the population who consume the drugs annually [6]. The WHO considers the minimum effective epidemiological coverage of the total population to be 65% [7]. More than five MDA rounds are required to bring infection levels below elimination thresholds in countries where drug coverage is poor [8]. MDA implementation requires collaboration and coordination of activities by national and local health office, nongovernmental organizations, communities, and donors.

Since 2000, annual MDA coverage has increased from 3 million people in 12 countries to 6.7 billion people in 66 of the 72 countries where LF is known to be endemic [5]. However, while the program has achieved effective coverage in several regions, it has had variable success in others [9]. High compliance of MDA is difficult to achieve and sustain. Individuals’ perceptions of the acceptability of interventions for LF elimination are being explored as a crucial component for their success, in addition to coverage and compliance [10]. Treatment acceptability refers to how likely people are to think an intervention is appropriate based on their cognitive or emotional responses to it [11].

The treatment’s importance, intrusiveness, characteristics, effectiveness, side effects, and whether it corresponds with the evaluator’s values or views are all regarded as contributing to the individual’s evaluations of its acceptability [11,12,13,14]. Individuals and communities who find MDA to be unacceptable are unlikely to want to participate in the intervention. Hence, in this review, we aim to present and systematically analyze the factors associated with the acceptability of mass drug administration (MDA) for filariasis together with future recommendations for accelerating the elimination program.

## 2. Materials and Methods

This systematic review is prepared in accordance with the PRISMA (Preferred Reporting Items for Systematic Reviews and Meta Analyses) updated guideline [15]. The objective of this review is to identify the factors associated with the acceptability of MDA for filariasis. The component of mnemonic PEO [16] (population, exposure, outcome) were established as follows:

- Population: general population.
- Exposure: mass drug administration for filariasis.
- Outcome: associated factors of acceptability.

### 2.1. Searching Strategy

The literature search was conducted in April 2022, using Web of Science, PubMed, and Scopus databases. The following were keywords used for searching of related articles: “associated factor*” OR “predictor” OR “determinant” OR “correlate*” AND “acceptability” OR “acceptance” OR “uptake” AND “mass drug administration” OR “MDA*” OR “ivermectin” OR “albendazole” OR “diethylcarbamazine” OR “triple-drug treatment” OR “triple-drug regime” OR “triple drug therapy” OR “IDA” AND “filariasis” OR “elephantiasis” OR “lymphatic filariasis”. All retrieved articles were imported into EndNoteX7 library, and library de-duplication was implemented according to Bramer et al. [17].

### 2.2. Eligibility Criteria

The inclusion was: (1) publication in the English language; (2) original articles including cohort, case–control, and cross-sectional as well as mixed methods and qualitative studies investigating the associated factors for acceptability of MDA. In contrast, non-original articles such as conference proceedings, perspective, commentary, opinion, reports, systematic review, and meta-analyses were excluded. Since GPELF was launched in 2000, which comprises annual MDA, the publication period was decided from 2000 onwards.

### 2.3. Study Selection

Four pairs of independent reviewers screened the titles and abstracts of the retrieved materials against the inclusive criteria. The potential articles identified during the main screening were kept, and the full text was reviewed independently by the same reviewers in detail according to the inclusive criteria. The third reviewer was assigned to resolve any disagreements that arose between each pair of reviewers.

### 2.4. Critical Appraisal and Data Extraction

Quality appraisal was conducted using the Mixed Method Appraisal Tool (MMAT). The MMAT evaluates the quality of qualitative, quantitative, and mixed-method studies. It focuses on methodological criteria and includes five core quality criteria for each of the following five categories of study designs: (1) quantitative, (2) qualitative, (3) randomized controlled, (4) nonrandomized, and (5) mixed methods [18]. One reviewer extracted the data, which were then assessed independently by the second reviewer.

### 2.5. Data Analysis

The study designs and reported outcomes varied significantly, so a meta-analysis could not be conducted on all included studies. Studies were excluded from the meta-analysis if the reviewers considered them to be inadequate for contributing meaningfully to the body of evidence. The uptake of MDA treatment and its 95% confidence intervals (CIs) were calculated using the initial number of eligible participants included and the number of the outcomes of interest (uptake of MDA treatment). Pooled estimates were derived using either random-effects or fixed-effects methods, depending on whether or not significant heterogeneity (defined as I^2^ > 30%) was present. The analyses were conducted using the statistical package ‘dosresmeta’ in R statistical software version 4.2.1 (Robert Gentleman and Ross Ihaka from Statistic Department of the University of Auckland, Auckland, New Zealand).

## 3. Results

**Figure 1:**
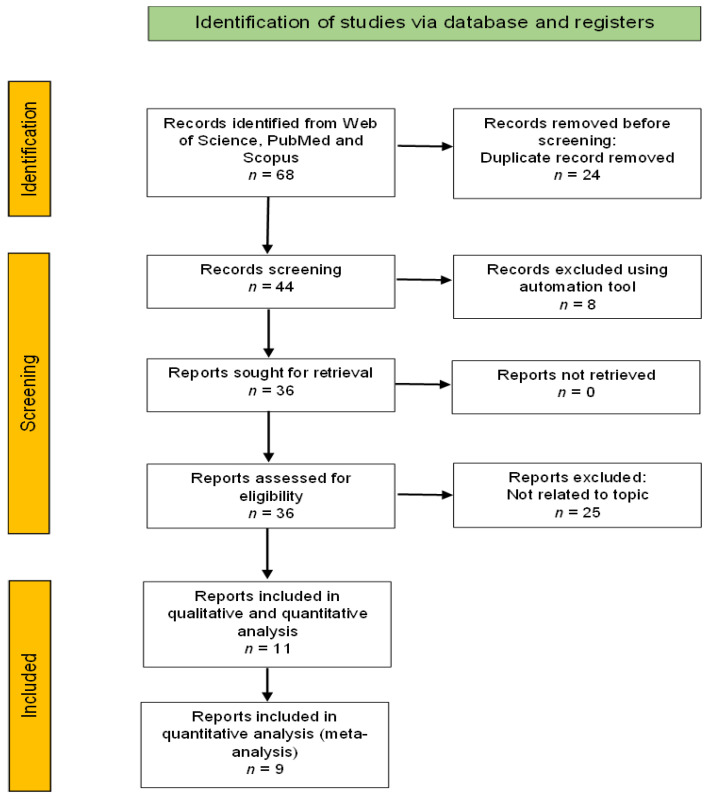
PRISMA flow diagram for the systematic review.

The search yielded 23 articles from SCOPUS, 25 from WOS, and 20 from PubMed, resulting in 68 unique hits. Only 11 articles were included in the full-text assessment after rigorous selection screening, as shown in the PRISMA flow diagram. Out of 11 articles, only 9 articles were included in the meta-analysis (Figure 1). A descriptive summary of the included studies in this review regarding study location and design is presented in Table 1. The findings from 11 studies were included in this systematic review, as shown in Table 2. Three eligible articles were from Indonesia, two from India, two from Tanzania, and one each from Guyana, Haiti, Nigeria, and the Philippines. The analyzed articles were published between 2004 and 2020. Four articles were cross-sectional studies, one article was a case–control study, and one was a longitudinal study. Three articles were qualitative studies, and another two used a mixed-method approach.

### 3.1. Acceptability of Mass Drug Therapy

In this article, 11 studies focused on MDA’s acceptability. Factors that facilitate the acceptance of MDA are divided into knowledge and awareness, attitude and perception, communication, access and delivery, and gender. Most of the studies included in this review show positive MDA acceptance. A study by Rosanti et al. [20] shows the highest drug compliance rate of 86.8%, followed by Bhatia et al. [23] with 77.7% and Putri et al. [22] with uptake of 75%. Meanwhile, the lowest compliance rate is illustrated by a study conducted by Nujum et al. [24] with 39.52%. Apart from that, factors that hinder the acceptability of MDA are lack of awareness, fear of side effects, refusal, laziness and unfavourable provider and client status toward the need for an MDA program.

Of eleven studies, three used DEC as a single drug [19,22,23], four used DEC and Albendazole [20,21,24,25], one used a triple-drug [9], one used Albendazole with either DEC or ivermectin and only one study mentioned MDA as a general and not the specific regime [19].

### 3.2. Factors Associated with Acceptability of Mass Drug Administration (MDA) for Filariasis

#### 3.2.1. Knowledge, Attitude and Perception

Knowledge and awareness of the disease itself were positively associated with acceptance and MDA participation compliance [9,24,25]. MDA acceptance was more likely to occur if villagers were familiar with the risks and benefits of MDA and its rationale for MDA [23].

Acceptance of the MDA was linked to a fear of filariasis, the desire to enhance one’s health and a trust in the intervention [27]. On the other hand, fear of certain MDA program elements, concerns about actual and perceived adverse effects of the medicine, led to rumours associated with lower acceptance of MDA for filariasis. [27]. Religious and traditional practises hampered MDA acceptance, as evidenced by low programme acceptance. A community in Ogun reported relying on prayer or home remedies to prevent disease, particularly when it was attributed to nonmedical causes. [19]. Unfavourable provider and client attitude toward the need for MDA was associated with noncompliance [24].

#### 3.2.2. Communication, Delivery and Access of the MDA

Community leaders and associated structures such as religious groups, village health committees and market associations were critical in shaping how communities responded to MDA [19]. Moreover, a high level of engagement with the target population increases people’s capacity to make informed choices, increasing uptake and acceptance [26]. However, language barriers and cultural variation become challenges in delivering messages, leading to low acceptance of MDA [19].

Difficulties in accessing and delivering medicine were due to gender role inequality [19]. Support from the administrator, local head community and TPE support (elimination executing staff) is associated with good acceptability of MDA [19,22]. Individuals with a history of drug uptake before showed better acceptance of MDA [25].

#### 3.2.3. Gender and Age

Gender relations emerged as a critical theme in access, uptake and compliance with MDA because they inherently affect decisions taken within the household regarding health [21]. Mathieu et al. found that men were likelier to have taken the drugs than women, and older people were approximately 30–40% more likely to take drugs than younger people [25].

#### 3.2.4. Risk of Bias

The authors conducted quality appraisal of all 11 studies using the Mixed Method Appraisal Tool (MMAT) [18]. The methodology quality of three categories of studies (qualitative study, quantitative descriptive study and mixed-methods study) can be appraised using this tool. For each category, five criteria are used to assess the quality of the study. The details of this assessment for the studies selected are reported in Table 3.

#### 3.2.5. Meta-Analysis

**Figure 2:**
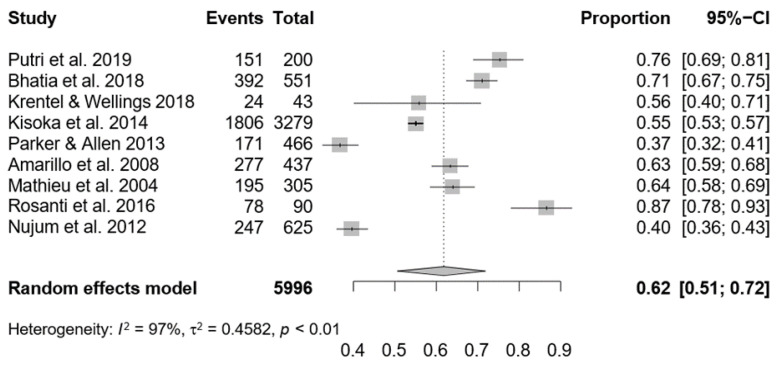
Forest plot of meta-analysis of uptake rate of MDA. (Putri et al. 2019 [22], Bhatia et al. 2018 [23], Krentel & Wellings 2018 [21], Kisoka et al. 2014 [25], Parker & Allen 2013 [26], Amarillo et al. 2008 [27], Mathieu et al. 2004 [28], Rosanti et al. 2016 [20], Nujum et al. 2012 [24]).

Only 9 of the 11 studies on MDA uptake have sufficient data to be used in a meta-analysis. The remaining 2 studies were unsuitable for meta-analysis due to the nonreporting of the required outcomes. The meta-analysis of the factors involved was not possible due to insufficient data. The forest plot of the relevant studies is shown in Figure 2. The pooled uptake rate of MDA is 62% (95% confidence interval (0.51–0.72). Heterogeneity was assessed with I^2^ or *p*. A *p*-value of ≤ 0.05 and I^2^ ≥ 50% were considered high heterogeneity. In this analysis, heterogeneity was noted to be high.

## 4. Discussion

A few factors contributed to the acceptability of the filariasis drug administration. They were classified into three categories: (1) knowledge, attitude and perception; (2) communication, delivery and access; and (3) age and gender.

### 4.1. Knowledge, Attitude and Perception

Knowledge is a potent tool with the potential to change people’s lives. To successfully control or eliminate a disease, the population involved must have prior knowledge of the disease and the treatment. In this study, knowledge of the MDA programme and the disease itself increased community participants’ acceptance of drug intake. This echoed a study performed in Egypt in which knowledge regarding the disease was associated with better drug compliance [29]. This is merely due to the good effort of the Egypt Ministry of Health in investing their budget in electronic media to promote the LF elimination programme [30]. Another study in Pakistan also showed that patients’ adherence to prescriptions was affected by their lack of understanding about the condition and the treatments used to treat it [31].

However, a study in Malaysia showed that the knowledge regarding filariasis is still low in even in areas endemic for the disease [32]. This was most likely due to a lack of awareness and health education programmes, as some of the endemic areas in Malaysia were in rural areas that were difficult to access via main roads [32,33]. Furthermore, knowledge transfer took place mostly in school programmes and may not have reached the entire community, particularly adults. As a result, the government should take a more comprehensive approach, such as publicizing information about filariasis through social media, television and radio, to help spread the word. However, Cabral et al. [12] found that despite the community’s good knowledge of filariasis, it did not seem to affect their low compliance with the MDA. This demonstrated that knowledge without a positive attitude is insufficient to increase acceptance or compliance with drug use.

Unfavourable provider and client attitude reduce drug acceptability. These findings support previous research that found that health personnel who offer detailed instructions on how to take medicine boost medication adherence and patient satisfaction [34]. According to an Indonesian study, respondents who were personally visited in their homes by health professionals had much higher medication adherence rates than those who were not. Door-to-door health personnel visits will raise community awareness of the importance of taking lymphatic filariasis medicine [35].

In this study, fear of the disease played a major role in increasing the acceptability of the drug uptake to treat the disease. A study in Kenya and India showed that communities which observe the presence of lymphatic filariasis patients in their midst know that everyone is in danger of developing lymphatic filariasis and are more willing to take the treatment. Furthermore, a person’s desire to engage in the next MDA programme was impacted by their belief that they also could have lymphatic filariasis [36,37]. Fear of actual and perceived medicine, fear of side effects, religious beliefs, and traditional remedies, on the other hand, reduce drug intake acceptance. Hussain et al. [37] stated that despite strong coverage and the fact that most adverse effects were moderate and uncommon, the fear of side effects was a big concern. A brief message detailing the most common, moderate side effects, as well as basic tips on how to manage them, might help to reduce fear and hence boost MDA compliance [37].

Certain patients firmly supported the use of traditional remedies for the treatment of their diseases, stating that they only switched to prescribed medicine given by health care personnel if conventional cures failed [38] (Clement et al. 2007). Most patients reported that drugs have more negative and unpleasant side effects than positive ones. Even those who did not think this had the impression that long-term pharmaceutical use may lead to other physiological problems [31].

Religious belief could also be one of the reasons for reduced compliance with the drug intake. A study in Ghana’s Bole district found that delivering medications during the Muslim fasting period made it impossible for most people to swallow the treatment, resulting in a drop in MDA coverage [39]. Another study in Papua New Guinea showed that supernatural beliefs, as well as a lack of awareness of the disease transmission model, had impacts on participation in disease prevention and treatment initiatives for lymphatic filariasis [40]. To improve MDA updates, health care personnel must be more aware of and sensitive to the culture and religious obligations of the local community.

### 4.2. Communication, Delivery and Access of the MDA

Good communication between leaders and villagers/community will increase filariasis drug uptake. A study in Ghana showed that when the community participation in mass drug administration approach fails, misconceptions and rumours about the programme spread like wildfire, obstructing execution [41]. In the past, a number of MDAs in various nations have experienced community scepticism. There are allegations that the medicines are used to poison minors, that they are used as birth control and that they cause erectile dysfunction [42]. By including traditional and religious leaders in these initiatives, social mobilization activities might help generate more engagement among community members [43].

When there is a difference in native language, a language or communication barrier usually occurs between patients and medical personnel. This could result in misinterpretation and miscommunication during drug administration. Miscommunication between doctors and patients is common, and it has a negative impact on the quality of care and patient satisfaction [44]. According to a study conducted in Indonesia, community empowerment is critical in improving drug acceptance in the community [45]. The information was delivered in local languages and was easily understood by the community, thus increased the acceptability of drug intake among community in the area [45].

### 4.3. Age and Gender

Men were found to be more likely to use the drug than women in this study. This is in agreement with a study in Egypt that showed that men may be more obedient in adhering to filariasis medication than women due to their increased understanding of the disease [29]. A study in India explained that this disparity might be because males have a greater literacy than girls [37]. A study in Taiwan came to the same conclusion that male patients are more likely to adhere to the medication compared with their female counterparts [46]. Adherence can be difficult for those with long, complicated prescription regimens, which could contribute to lower adherence among women, who are more likely to take many medications [47]. It is also probable that female patients are more likely to have drug adverse effects [48].

Women may also face different expectations and priorities that may influence how much attention they pay to their own health. Women are responsible for caring for others in their households, and as a result, they may neglect their own needs [49]. A negative attitude toward drugs has been linked to poor adherence, with women being more negative than men. Some women have experienced more severe adverse medication reactions than men, which may have influenced the gender difference. [50]. The frequent media coverage of drug-related concerns while pregnant or breastfeeding may make women feel more vulnerable to drug risks in general and fear the development of an adverse drug reaction. For example, pregnancy has been identified as a leading cause of medical treatment discontinuation. [51,52]. Another reason could be due to safety reasons where the male drug distributors cannot administer to women in the household without the attendance of the male family members [53].

However, this is in contrast with a study in Indonesia, in which men who worked were aware that filariasis medications could have side effects that could wreak havoc on their health and as a result their finances and were therefore less likely to take anti-filariasis medications because they did not want their jobs disturbed [54]. In another study, conducted in Uganda, women were perceived more accepting of drug use [55]. This was likely the result of a lack of strategy during the house-to-house drug distribution programme, during which men were more likely to be outside the home due to occupational requirements. Women, on the other hand, were more likely to receive treatment as they spent most of their time at home doing domestic chores and, in turn, may have had more knowledge of the programme due to their contact with the health provider [55,56].

In this study, older people were more likely to take the drug than younger people. This is similar to a previous study in Egypt that showed that younger people had more awareness of lymphatic filariasis than the general community but they also had lower MDA compliance rates as they may have been more likely than others to be absent when the drugs were distributed [29]. Older patients also tend to have more severe illnesses than younger patients, which raises their awareness of their health situation, which appears to have a favourable influence on adherence [57]. Older people are more likely to be exposed to health-related programmes and to have grasped the benefits of engaging in them [58].

Teenagers adhere to therapies with more immediate and possibly catastrophic repercussions if they are not followed, as opposed to those with less clear benefit or more intrusive to their lifestyle [59]. Young adults will believe primarily what they can see or have experienced, and hence are unable to fully grasp the long-term or unknown effects of failing to take their drugs. They have a much greater burden since they frequently lack fully developed risk assessment, impulse control, and organizational skills [59]. However, this is in contrast to a study conducted in Pondicherry, South India, that showed filariasis drug-taking behaviour was considerably lower among respondents aged 61 and up [60]. A study in Iran also found that drug use and adherence are poor in the nation, particularly among the elderly, and that efforts to improve them have been ineffective [61]. Adherence was also inversely correlated with multimorbidity and cognitive impairment [62]. Hence, it is important to target older patients with multimorbidity and cognitive impairment for adherence treatments, to enhance awareness about therapy and simplifying regimens.

### 4.4. Recommendation

Knowledge and awareness regarding LF and its treatment have been identified as important factors in increasing the acceptance of MDA. Therefore, specific attention must be made to communities with less knowledge and awareness. Nevertheless, these factors should not be treated in isolation, as it is also important to consider other factors which motivate the acceptance of MDA [63]. This review also identified provider and client attitude as important factors which can influence the acceptance of MDA in the community. In MDA, the final interplay is between client and drug administrator. The outcome depends on how competent the drug administrators are, and therefore, they should be well trained in order to convey the messages regarding the need of MDA. The role of health system factors including the training of health workers and drug distributors was also demonstrated in a study conducted in India [64]. Clients’ unfavourable attitudes towards MDA could be due to poor understanding regarding the role of MDA in interrupting transmission and disease elimination. Hence, health education should focus on the need for MDA in order to save the next generation from this dreadful disease [24].

Fear of side effects is one of the main reasons which hinder the uptake of MDA. The fear of side effects in the community should be addressed during community mobilization activities, and messages regarding the positive aspects of adverse reactions should be carefully incorporated in health communication campaigns. Furthermore, there is a need to develop active surveillance systems for detecting and managing adverse reactions during MDA [65]. Religious and traditional beliefs were among the challenges in improving the acceptance of MDA. This finding indicates the need for awareness and sensitization activities to respond to syncretic belief systems in order to allow individuals to make appropriate informed decisions about accepting MDA [66].

As emphasized above, community engagement is essential for increasing the acceptability of MDA. Given that it is the economically disadvantaged individuals who are mostly infected with neglected tropical diseases including filariasis, there is a need for engagement with such populations and for developing local-level strategies for improving the acceptance of MDA [66]. Additionally, language barriers and cultural variations must be addressed. For instance, strategies may be used to increase the uptake of MDA for example, tailoring service by providing information and services in local languages or making it more sensitive to cultural and religious beliefs, including gender norms [67].

Gender relations can influence the uptake of MDA as they can affect the decisions regarding health in the household. Therefore, health interventions must consider the complexity of gender roles. Both men and women in a family must be approached to improve the uptake of MDA [21]. Additionally, the provision of culturally sensitive services such as services to women by female health providers can be used for increasing the acceptance of MDA in females [67]. This review also identified age as a factor that contributes to the acceptance of MDA. In communities where LF is transmitted, all ages are affected. When the infection occurs during childhood, visible manifestations such as limb oedema may occur later in life, leading to disability. Based on the WHO recommendations, except for children below two years old, individuals of all ages in the population at risk of LF transmission are eligible for MDA [67]. Hence, the MDA programmes need to be strengthened in order to reach all age groups.

### 4.5. Limitation

As with any research, this systematic review is not without limitations. Even though 47 countries worldwide remain threatened by LF and require preventive chemotherapy [2], we only identified articles from 7 countries. Moreover, the role of publication bias in this systematic review must be acknowledged as grey literature was not included. Furthermore, language bias should also be considered as we only included articles published in English, although our search strategy resulted in literature sourced from several countries where English is not the primary language (Indonesia, Tanzania, Haiti). Despite these limitations, this systematic review synthesizes research evidence regarding the factors associated with the acceptance of MDA in communities, which may serve as a guide to improving service delivery strategies of the MDA.

## 5. Conclusions

To meet elimination targets, MDA coverage must be expanded in order to cover all the endemic countries. Nevertheless, despite the increase in MDA coverage, community acceptance remains a challenge in the implementation of MDA. Therefore, understanding the factors influencing the acceptance of MDA, as highlighted in this review, is critical. These findings may be utilized to improve the implementation of MDA, which would help to maximize the acceptance in the community and contribute to the successful elimination of LF.

## Figures and Tables

**Table 1:** Summary of study location and study design.

**Study Location**	**Authors**
Guyana	Niles et al. 2021 [9]
Nigeria	Adekeye et al. 2020 [19]
Indonesia	Rosanti, Mardihusodo, & Artama 2016., Krentel & Wellings 2018, Putri et al. 2019 [20,21,22]
India	Bhatia et al. 2018, Nujum et al. 2012 [23,24]
Tanzania	Kisoka et al. 2014, Parker & Allen 2013 [25,26]
Philippines	Amarillo et al. 2008 [27]
Haiti	Mathieu et al. 2004 [28]
**Study Design**	**Authors**
Cross-sectional	Niles et al. 2021, Putri et al. 2019, Kisoka et al. 2014, Mathieu et al. 2004 [9,22,25,28]
Longitudinal study	Rosanti, Mardihusodo, Artama 2016 [20]
Case-control	Nujum et al. 2012 [24]
Qualitative study	Adekeye et al. 2020, Krentel et al. 2018, Parker & Allen 2013 [19,21,26]
Mixed method	Bhatia et al. 2018, Amarillo et al. 2008 [23,27]

**Table 2:** Summary of accepted articles.

Author (Year)	Title	Study Design	Sample Size	Types of Drug Therapy/Regime	Acceptability	Factors
Niles RA et al. 2021 [9]	Assessing factors influencing communities’ acceptability of mass drug administration for the elimination of lymphatic filariasis in Guyana	Cross-sectional	390	Triple drug therapy regimen IDA (Ivermectin, DEC, and Albendazole	Intervention Rating Profile tool. Mean acceptability scores ranged from 24.6 to 29.3, above the threshold of acceptability (a score of 22.5).	1. Region 2. Knowledge 3. Compliance Regional variation occurred across many indicators of interest: self-rated understanding about LF, mechanisms of LF transmission, LF drug safety, and history of treatment during MDA
Adekeye et al. 2020 [19]	Mass administration of medicines in changing contexts: Acceptability, adaptability and community-directed approaches in Kaduna and Ogun states, Nigeria	Qualitative study	42	Ivermectin		1. Belief 2. Perception about medicines and their side effects 3. Community leaders and associated structures such as religious groups, village health committees and market associations were critical in shaping how communities responded to MDA
Putri et al. 2019 [22]	Factors determining drug uptake during mass drug administration in Banyuasin district, South Sumatera, Indonesia	Cross-sectional	200	Combination of DEC 6 mg/kg body weight, Albendazole 400 mg and Paracetamol 500 mg given once a year for a minimum of 5 consecutive years	Uptake rate 75.5%	1. Attitudes 2. Support from elimination executing staff
Bhatia et al. 2018 [23]	Mass drug administration (MDA) for the elimination of lymphatic filariasis: Experiences from Nayagarh district of Odisha, India	Mixed method	551	DEC and Albendazole	Drug compliance rate (77.7%) Coverage compliance gap (22.3%)	Barriers for uptake are 1. Low level of awareness of the benefits of MDA 2. Fear of side effects due to the treatment, 3. Low confidence in the MDA program 4. Inadequate persuasion
Krentel & Wellings 2018 [21]	The role of gender relations in uptake of mass drug administration for lymphatic filariasis in Alor District, Indonesia	Qualitative Study	43	A single dose of DEC or Ivermectin (in those areas where onchocerciasis or loiasis is endemic) in combination with Albendazole	24/43 compliant treatment (55.8%) 19/43 non-compliant (44.2%)	Gender relations emerged as a key theme in the access, uptake, and compliance with MDA. Four models of responsibility for health decision-making emerged: (i) responsibility resting primarily with the husband. (ii) responsibility resting primarily with the wife. (iii) responsibility shared equally by husband and wife; and (iv) responsibility autonomously assumed by everyone for his or her own self, regardless of the course of action of the other spouse
Kisoka et al. 2014 [25]	Factors influencing drug uptake during mass drug administration for control of lymphatic filariasis in rural and urban Tanzania	Cross-sectional	3279	Combination of Ivermectin (150–200 µg/kg body weight) and Albendazole (400 mg)	Overall drug uptake rate was 55.1% (range of 44.5–75.6% between districts)	Factors associated with high uptake: 1. Increasing age 2. History of previous drug intake Factors associated with low uptake: 1. Absent from home during drug distribution 2. Clinical contraindication to the treatment 3. Missing household visit of drug distributors 4. Household not being informed about the drug distribution
Parker & Allen 2013 [26]	Will mass drug administration eliminate lymphatic filariasis? evidence from Northern Coastal Tanzania	Qualitative Study	108 villagers	Albendazole, in combination with either DEC or Ivermectin;	Mwembeni village uptake 2007: 306 (34%) Jaira village uptake in 2007: 160 (42%)	Factors associated with low uptake: 1. Fear of treatment (questioning the motives behind free drugs by the government, fear of side effects, doubt of drug efficacy, lack of knowledge); 2. Divergence between biomedical understanding of lymphatic filariasis and local understanding of swollen scrotum (mabusha) and swollen limbs (matende) (belief that these two symptoms are related to sexual intercourse, acts of God, witchcraft, etc.); 3. Limited and ineffective communication (few people understood the rationale for distributing the drugs for free) 4. Too great a reliance on voluntary drug distributors (those living far away were not reached) Factors associated with high uptake: 1. High level of engagement with the target population resulting increased capacity of people to make informed choices (those living in town, those who had been visited by medical staff and researchers)
Amarillo et al. 2008 [27]	Factors associated with the acceptance of mass drug administration for the elimination of lymphatic filariasis in Agusan del Sur, Philippines	Mixed method	437	DEC and Albendazole	Acceptance rate: 60%	1. Moderate knowledge of lymphatic filariasis 2. High perceived benefits of antifilarial drug 3. Awareness of lymphatic filariasis 4. Awareness of MDA 5. Awareness of MDA for lymphatic filariasis
Mathieu et al. 2004 [28]	Factors associated with participation in a campaign of mass treatment against lymphatic filariasis in Leogane, Haiti	Cross-sectional	305	DEC and Albendazole	MDA coverage: 63.9%	1. Male gender (OR = 3.3; CI = 1.5–7.4) 2. Knowledge that filariasis is mosquito-borne (OR = 2.6; CI = 1.2–5.4) 3. Having received a filariasis-related health-education message through posters and banners (OR = 2.9; CI = 1.2–7.5)
Rosanti et al. 2016 [20]	Directly observed treatment increases drug compliance in lymphatic filariasis mass drug administration	Longitudinal study	90	A single dose of DEC (three 100 mg tablets for persons weighing 50 kg) with the addition of a single 400 mg dose of Albendazole	Drug compliance rate was 86.80%	Reasons for failing to take drugs: 1. Fear of side effects (50%) 2. Refusals (25%) 3. Laziness (16.7%) 4. Perceiving the drug to be useless (8.3%) 5. Drug compliance observer (non-family)
Nujum et al. 2012 [24]	Factors determining noncompliance to mass drug administration for lymphatic filariasis elimination	Case control	99 cases (non-compliant), 70 control (compliant)	Single dose DEC	39.52% taken the drug (247/625)	Non-compliant status associated with 1. Unfavourable provider attitude toward the need of the program 2. Unfavourable client attitude towards the need of the program 3. Low drug administrator acceptability

**Table 3:** The details of the MMAT assessment.

Author	Type of Study	1.1	1.2	1.3	1.4	1.5
		Is the sampling strategy relevant to address the research question?	Is the sample representative of the target population?	Are the measurements appropriate?	Is the risk of nonresponse bias low?	Is the statistical analysis appropriate to answer the research question?
Niles RA et al. 2021 [9]	Quantitative descriptive	Yes	Yes	Yes	Yes	Yes
Putri et al. 2019 [22]	Quantitative descriptive	Yes	Yes	Yes	No	Yes
Kisoka et al. 2014 [25]	Quantitative descriptive	Yes	Yes	Yes	Can’t tell	Yes
Mathieu et al. 2004 [28]	Quantitative descriptive	Yes	Yes	Yes	Yes	Yes
Rosanti et al. 2016 [20]	Quantitative descriptive	Yes	Yes	Yes	Yes	Yes
Nujum et al. 2012 [24]	Quantitative descriptive	Yes	Yes	Yes	Yes	Yes
		Is the qualitative approach appropriate to answer the research question?	Are the qualitative data collection methods adequate to address the research question?	Are the findings adequately derived from the data?	Is the interpretation of results sufficiently substantiated the data?	Is there coherence between qualitative data sources, collection, analysis and interpretation?
Adekeye et al. 2020 [19]	Qualitative	Yes	Yes	Yes	Yes	Yes
Krentel & Wellings 2018 [21]	Qualitative	Yes	Yes	Yes	Yes	Yes
Parker & Allen 2013 [26]	Qualitative	Yes	Yes	Yes	Yes	Yes
		Is there an adequate rationale for using a mixed methods design to address the research question?	Are the different components of the study effectively integrated to answer the research question?	Are the outputs of the integration of qualitative and quantitative component adequately interpreted?	Are the divergences and inconsistencies between quantitative and qualitative results adequately addresses?	Do the different components of the study adhere to the quality criteria of each tradition of the methods involved?
Amarillo et al. 2008 [27]	Mixed-method	Yes	No	No	No	No
Bhatia et al. 2018 [23]	Mixed-method	Yes	Yes	Yes	No	Yes

## Data Availability

The data that support the findings of this study are available from the corresponding author upon reasonable request.
